# Biomechanical function of the anterolateral ligament of the knee: a systematic review

**DOI:** 10.1186/s43019-019-0021-3

**Published:** 2020-01-01

**Authors:** Jin Kyu Lee, Young Jin Seo, Soo-Young Jeong, Jae-Hyuk Yang

**Affiliations:** 1 Department of Orthopaedic Surgery, Hanyang University Hospital, Seoul, South Korea; 2 Department of Orthopaedic Surgery, Dongtan Sacred Heart Hospital, Dongtan, Gyeonggi-do South Korea; 3 Department of Orthopedic Surgery, Guri Hospital, Hanyang University Medical Center, 153, Gyeongchunro, Guri, Gyeonggi-Do 11923 South Korea

**Keywords:** biomechanical function, anterolateral ligament, knee, systematic review

## Abstract

**Background:**

It has been suggested that the anterolateral ligament (ALL) is an important anterolateral stabilizer of the knee joint which functions to prevent anterolateral subluxation and anterior subluxation at certain flexion angles in the knee.

**Purpose:**

To analyze and systematically interpret the biomechanical function of the ALL.

**Methods:**

An online search was conducted for human cadaveric biomechanical studies that tested function of the ALL in resisting anterolateral subluxation and anterior subluxation of the knee. Two reviewers independently searched Medline, Embase, and the Cochrane Database of Systematic Reviews for studies up to 25 September 2018. Biomechanical studies not reporting the magnitude of anterior tibial translation or tibial internal rotation in relation to the function of the ALL were excluded.

**Results:**

Twelve biomechanical studies using human cadavers evaluating parameters including anterior tibial translation and/or internal tibial rotation in anterior cruciate ligament (ACL)-sectioned and ALL-sectioned knees were included in the review. Five studies reported a minor increase or no significant increase in anterior tibial translation and internal tibial rotation with further sectioning of the ALL in ACL-deficient knees. Five studies reported a significant increase in knee laxity in tibial internal rotation or pivot shift with addition of sectioning the ALL in ACL-deficient knees. Two studies reported a significant increase in both anterior tibial translation and internal tibial rotation during application of the anterior-drawer and pivot-shift tests after ALL sectioning.

**Conclusion:**

There was inconsistency in the biomechanical characteristics of the ALL of the knee in resisting anterolateral and anterior subluxation of the tibia.

## Introduction

The existence of a specific structure in the knee’s lateral capsule was discovered by dissections performed in 1879 by Paul Segond. He described it as “a resistant, pearly, fibrous band, which, in an exaggeration of internal rotational movement, is always subjected to an extreme degree of tension” as well as an avulsion fracture now named the “Segond fracture.” [1] This “recently” described structure was named the “anterolateral ligament” (ALL) by Vieira et al. [2] in 2007. The ALL has been the subject of many recent publications although there has not always been agreement with each other in the anatomic origins [3, 4].

Despite recent improvements in surgical methods and understanding of ACL anatomy, it has been suggested that the normal rotational stability of the knee is not fully restored by reconstructive methods for ACL injuries [5, 6]. Such abnormal biomechanics have led surgeons to focus more on anterolateral structures and, in the past few years, the ALL of the knee has been studied with regard to its anatomy and biomechanics [3, 7–9].

Although several biomechanical studies have been published that the ALL is an important anterolateral stabilizer of the knee joint that prevents anterolateral subluxation (internal tibial rotation) and anterior subluxation at certain flexion angles in the knee [7, 10–12], because of the variability in anatomic descriptions and methodology in biomechanical testing, some concepts regarding the biomechanical function of the ALL are controversial.

Given the relative paucity of literature reviewing the native biomechanics of the ALL of the knee, the purpose of the present study was to provide a systematic review of the biomechanical ALL function excluding the surgical aspect of ACL and/or ALL. The hypothesis of this study was that there would be inconsistent results in the biomechanical characteristics of the ALL of the knee in resisting anterolateral and anterior subluxation of the tibia.

## Methods

### Identification and selection of articles

Two reviewers independently searched Medline, Embase, and the Cochrane Database of Systematic Reviews for studies up to 25 September 2018. The following search protocol was used: “anterolateral ligament” [All Fields] OR “anterior lateral ligament” [All Fields] OR “ALL” [All Fields] OR “Segond fracture” [All Fields] OR “lateral capsular ligament” [All Fields]) OR “anterior oblique band” [All Fields]) OR “iliotibial tract” [All Fields] AND “biomechanical study” [All Fields]) OR “biomechanical phenomena” [All Fields]) OR “biomechanical phenomena” [All Fields] OR “phenomena, biomechanical” [All Fields] OR “kinematics” [All Fields] OR “biomechanical assessment” [All Fields]) OR “biomechanical function” [All Fields]) OR “biomechanically” [All Fields]) OR “cadaver” [Mesh] OR “cadaver” [All Fields] OR “cadavers” [All Fields] OR “human cadaveric study” [All Fields]) OR “cadaver study” [All Fields].

The inclusion criteria were English language, human cadaveric study, and biomechanics or biomechanical studies on the function of the ALL of the knee. The exclusion criteria were studies on (reconstructive or tenodesis) surgical outcomes; anatomic and radiographic studies of the ALL of the knee; biomechanical studies not reporting the magnitude of anterior tibial translation or tibial internal rotation; review or commentary articles. Duplicated articles were excluded, and two independent reviewers studied the abstracts from all searched articles. After initial identification of articles, full-text review of the selected studies was undertaken.

### Data collection

Study data were collected, specifically those detailing the: torque applied; sequence of the experimental protocol; magnitude of anterior tibial translation and/or internal tibial rotation; other relevant reported results. Quality assessment of the studies was not done because all biomechanical studies did not have associated level of evidence.

## Results

**Fig. 1:**
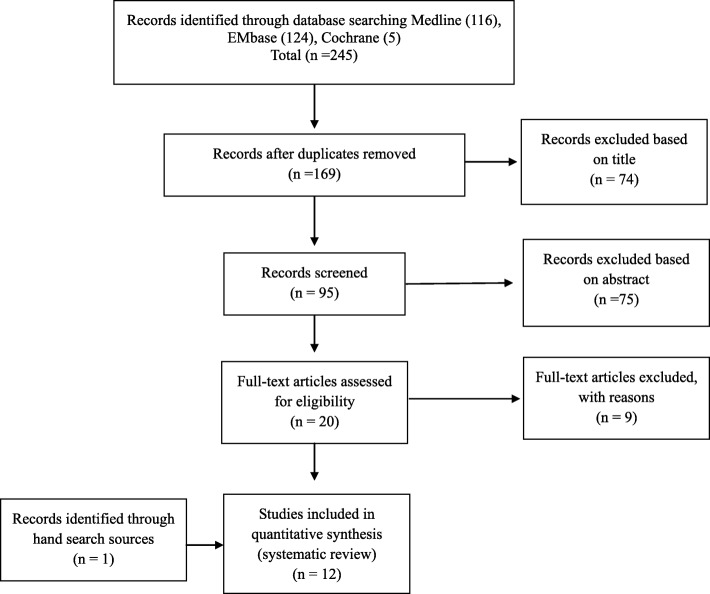
Preferred reporting items for systematic review flowchart showing application of selection criteria to the studies identified with the search strategy

The literature search identified 245 articles. Review of the titles and abstracts excluded 76 duplicates as well as 149 studies not related to biomechanical study of the ALL of the knee. This strategy left 20 articles for full-text review. Three studies focusing on the wrong topic, two studies related to the outcome of surgical reconstruction, and four studies not reporting the degree of anterior tibial translation and/or internal tibial rotation were excluded, thereby leaving eleven articles. A manual search for reference lists identified one additional article, so twelve articles were finally included for systematic review. (Fig. 1) Risk of bias assessment was done for each included study (Table 1).

**Table 1:** Risk of bias assessment for interrupted time series studies

	Intervention independent of other changes	Shape of the intervention effect pre-specified	Intervention unlikely to affect the data collection	Knowledge of allocated interventions adequately prevented during the study	Incomplete outcome data adequately	Selective outcome reporting	Other risks of bias
Noyes et al. 2017 [13]	L	L	L	H	U	L	L
Inderhaug et al. 2017 [10]	L	L	L	H	U	L	L
Drews et al. 2017 [14]	L	L	L	H	U	L	U
Sonnery-Cottet et al. 2016 [15]	L	L	L	H	U	L	L
Thein et al. 2016 [16]	L	L	L	H	U	L	L
Spencer et al. 2016 [17]	L	L	L	H	U	L	L
Ruiz et al. 2016 [12]	L	L	L	H	U	L	L
Rasmussen et al. 2016 [18]	L	L	L	H	U	L	L
Bonanzinga et al. 2016 [7]	L	L	L	H	U	L	U
Saiegh et al. 2015 [19]	L	L	L	H	U	L	U
Parsons et al. 2015 [20]	L	L	L	H	U	L	L
Monaco et al. 2012 [11]	L	L	L	H	U	L	L

*L* low risk of bias, *H* high risk of bias, *U* unclear risk of bias

Twelve biomechanical studies using human cadavers evaluating parameters including anterior tibial translation and/or internal tibial rotation in ACL-sectioned and ALL-sectioned knees are summarized in Table 2. Five studies (out of the 12 studies reviewed) reported a minor increase or no significant increase in anterior tibial translation and internal tibial rotation with further sectioning of the ALL in ACL-deficient knees [13, 14, 16, 17, 19]. The remaining 5 studies reported a significant increase in either the anterior translation and knee laxity in tibial internal rotation or pivot shift with addition of ALL sectioning in ACL-deficient knees [7, 10–12, 20]. Two studies reported a significant increase in both anterior tibial translation and internal tibial rotation during application of the anterior-drawer and pivot-shift tests after ALL sectioning [15, 18].

**Table 2:** Biomechanical studies reviewed

Study/year	Age (years)	Sequence of experimental protocol	Torque applied	Key findings
Noyes et al. 2017 [13]	44 ± 8	Intact-ACL section-ALL section-ITB section Intact-ACL section-ITB section-ALL section	IR (5 Nm), AD (100 N), AD+IR + VG (100 N + 5 Nm or 1 + 7 Nm)	ACL section alone produced increased PS and AD. Further section of ALL or ITB separately produced minor increase in AT (< 2 mm) and IR (< 3°). Concurrent section of ALL and ITB resulted in conversion to a grade-3 PS.
Inderhaug et al. 2017 [10]	57	Intact-ACL section-ALL section	IR (5 Nm), AD (90 N), AD+IR (90 N + 5 Nm)	Significant increase in AT and IR with additional section of ALL to ACL.
Drews et al. 2017 [14]	57	Intact-ACL section-ALL section	AD (134 N), VG + IR (10 Nm + 4 Nm)	ALL does not function under passive motion and with no influence on tibial rotation.
Sonnery-Cottet et al. 2016 [15]	76.4	Intact-ACL section-ALL section. On the contralateral limb, reverse order	IR (2 Nm) using the navigation system	ALL is involved in rotational control at varying degrees of knee flexion. Concomitant to an ACL or ITB transection, sectioning of ALL further increased rotational laxity.
Thein et al. 2016 [16]	43 ± 15	Intact-ACL section-ALL section	AD (134 N), VG + IR (8 N + 4 Nm)	Minor increase in AT (2–3 mm) in AD and PS with section of ALL to ACL.
Spencer et al. 2016 [17]	74 ± 12	Intact-ACL section-ALL section	AD (90 N), VG + IR (10 N + 5 Nm)	ALL section had no significant impact on AT in ACL-deficient knees. ALL section had significant impact only on early PS in ACL-deficient knees.
Ruiz et al. 2016 [12]	76	Intact-ACL section-ALL section Intact-ALL section-ACL section	IR (0–8 Nm)	IR increased significantly after each stage of section.
Rasmussen et al. 2016 [18]	49.3	Intact-ACL section-ALL section	AD+IR (88 N + 5 Nm), VG + IR (10 N + 5 Nm)	Significant increase in AT at 0°, 15°, 30°, and 60° of knee flexion, and in IR at all flexion angles when PS force applied after ALL section.
Bonanzinga et al. 2016 [7]	79 ± 5	Intact-ACL section-ALL section	AD (max manual), AD+IR + VR (manual)	No further increase in AT was found after ALL section. Significant increase of IR at 30° and 90° flexion only after additional ALL sectioning. ACL- and ALL-sectioned knees have significantly more acceleration of PS than that in intact knees.
Saiegh et al. 2015 [19]	42	Intact-ACL section-ALL section	AD (120 N), VG (40–50 N) + manual axial compression	No change in AT or IR after subsequent sectioning of ALL.
Parsons et al. 2015 [20]	76.3	Intact-ACL section-LCL section-ALL section	AD (134 N), IR (5 Nm)	ALL is important stabilizer of internal rotation at flexion angles greater than 35°. The ACL is the primary resister during anterior draw at all flexion angles and during internal rotation at flexion angles less than 35°
Monaco et al. 2012 [11]	72	Intact-ACL section-ALL section	AD (manual), AD+IR + VR (manual)	5.5° increase of IR at 30° flexion after additional ALL sectioning. No significant increase of AT after additional ALL sectioning.

*ACL* anterior cruciate ligament, *ALL* anterolateral ligament, *LCL* lateral collateral ligament, *ITB* iliotibial band, *PS* pivot shift, *IR* internal tibial rotation, *AD* anterior drawer, *VG* valgus, *AT* anterior tibial translation

## Discussion

This systematic review of the biomechanical studies of the ALL of the knee revealed inconsistencies due to following factors. First, the threshold value for statistical significance was different. If adopting a threshold value of < 3 mm for anterior tibial translation and < 2° for internal tibial rotation, as suggested by Noyes et al. [13], some of studies would have been interpreted differently. Second, the magnitude of torque applied and the position of the knees for simulating Lachman and pivot-shift tests were inconsistent. Five of the 12 studies did not demonstrate the significance of ALL both for anterior translation and internal tibia rotation [13, 14, 16, 17, 19]. While other 5 studies showed either the tibia internal rotation or anterior translation effect after ALL sectioning [7, 10–12, 20], 2 studies showed the statistical significance both in anterior translation and internal tibia rotation [15, 18]. In addition, management of ITB was different among studies.

Biomechanical studies included in this systematic review had a common experimental protocol with regard to the sequence of ligament sectioning. All biomechanical studies compared the degree of anterior tibial translation and/or internal tibial rotation after sectioning of the ACL followed by sectioning of the ALL [7, 10–20]. The main variation or inconsistencies between studies probably resulted from differences in the torque and maneuvers applied to the cadaveric knees. Universal protocols to simulate the pivot-shift and anterior-drawer tests of the knee are lacking, so such inconsistency may be inevitable.

Five studies (out of the nine reviewed) showed only a minor increase or no significant increase in anterior tibial translation or internal tibial rotation (i.e., pivot shift) with further sectioning of the ALL in ACL-deficient knees. Noyes et al. [13] found only a minor increase in the pivot shift and tibial internal rotations (< 2 mm or < 3°) after ALL or iliotibial band (ITB) sectioning in ACL-deficient knees. They concluded that anatomic ALL or ITB reconstructions would not block a positive pivot shift. However, concurrent sectioning of the ALL and ITB resulted in conversion in most knees to a grade-3 pivot-shift subluxation, suggesting that most biomechanical studies overestimated the function of the ALL by removing ITB. Saiegh et al. [19] also measured anterior tibial translation and internal tibial rotation after sectioning the ALL in ACL-deficient knees. They found increases of only − 0.7 mm and 0.3° in the anterior tibial translation and internal tibial rotation, respectively. ITB was also detached in this study. Spencer et al. [17] reported very similar outcomes with two prior studies, reporting an increase of only 2° in internal tibial rotation after additional ALL sectioning in ACL-deficient knees. ITB was not damaged in their study by incising the posterior border of longitudinal fibers and retracting anteriorly. It has been suggested that 99% of chronic ACL ruptures and 95% of acute ACL ruptures display a difference of ≥3 mm in anterior tibial translation, so 2 mm (< 3 mm) had been chosen as the threshold for a significant difference in previous studies [13, 21, 22]. Thein et al. [16] reported similar results to the previous studies because they found a mean increase of 2–3 mm (i.e., within the threshold value) in anterior tibial translation in anterior-stability and simulated pivot-shift tests. Furthermore, the load borne by the ALL in ACL-intact knees was minimal in response to simulated pivot-shift and anterior-drawer tests. They concluded that the ALL is a secondary stabilizer to the ACL whereby only the ALL bears increased loads at extremes of tibial translation in ACL-deficient knees. ITB was reflected in this series.

In contrast, five studies reported a significant increase in knee laxity either in tibial internal rotation or pivot shift with addition of ALL sectioning in ACL-deficient knees. Inderhaug et al. [10] found a significant increase in knee laxity by adding an anterolateral lesion to ACL-deficient knees when an anterior drawer force and internal tibial torque was applied. Furthermore, they identified a significant restoration of knee laxity when a combined lateral tenodesis and MacIntosh procedure were combined to ACL reconstruction. However, their study was limited because clinically significant threshold values were not applied. The ITB was transected in this series. Conversely, Monaco et al. [11] reported a significant increase in internal tibial rotation of 5.5° at a knee flexion of 30° after ALL sectioning, although there was no significant increase in anterior tibial translation after additional ALL sectioning. ITB was not damaged in their study by incising the longitudinal fibers and retracting anteriorly. Bonanzinga et al. [7] reported a significant increase in tibial internal rotation at 30° and 90° of knee flexion after additional sectioning of the ALL. They also found increased acceleration of the pivot shift if the ACL and ALL were sectioned compared with the intact state. The ITB was separated longitudinally. Ruiz et al. [12] applied only an internal rotation force without the anterior-drawer test, and reported a significant additional increase in internal tibial rotation regardless of the sequence of sectioning between the ACL and ALL. The ITB was incised longitudinally in this series.

Two studies reported a significant increase in both anterior tibial translation and internal tibial rotation during anterior-drawer and pivot-shift tests after ALL sectioning [15, 18]. Robotic biomechanical testing was undertaken, and a significant increase in anterior tibial translation was found at 0°, 15°, 30°, and 60° of knee flexion, and in internal tibial rotation at all flexion angles when the pivot shift force was applied [18]. However, similar to other studies, clinically significant threshold values were not applied. Management of ITB was not described in detail. Sonnery-Cottet et al. [15] tested the cadaveric knees in internal rotation at 20° and 90° of flexion and then subsequently tested using a simulated pivot-shift test consisting of coupled axial rotation at 30° of flexion. Serial sectioning of the ACL, ALL, and ITB was performed. After ACL sectioning, an incision of the ALL induced a significant increase in internal rotation (119.2% [*P* = .0002] at 20°; 121.8% [*P* = .0029] at 90°) and in coupled axial rotation (143.0%; *P* = .0035) compared with the intact knee as well as a significant increase in internal rotation at 90° (113.4%; *P* = .009) and in coupled axial rotation (130.8%; *P* = .0124) compared with the ACL deficient knee. They concluded that the ALL is involved in rotational control of the knee at varying degrees of knee flexion and during a simulated pivot shift. They also mentioned that the concomitant to an ACL or ITB transection, sectioning the ALL further increased rotational laxity.

This systematic review has a few limitations. First, the heterogeneous protocols and experimental settings of biomechanical studies and different threshold values limited the scope of direct comparisons for biomechanical results. Second, several biomechanical studies were excluded because of different assessment parameters and status of knees (i.e., surgically reconstructed). Tavlo et al. [23] undertook a similar biomechanical cadaveric study but, after ACL reconstruction, found no significant difference between an intact and sectioned ALL with regard to anterior tibial translation.

## Conclusion

There were inconsistent results among studies in the biomechanical characteristics of the ALL of the knee in resisting anterolateral and anterior subluxation of the tibia.

## Data Availability

Possible.
